# A study on a robot arm driven by three-dimensional trajectories predicted from non-invasive neural signals

**DOI:** 10.1186/s12938-015-0075-8

**Published:** 2015-08-20

**Authors:** Yoon Jae Kim, Sung Woo Park, Hong Gi Yeom, Moon Suk Bang, June Sic Kim, Chun Kee Chung, Sungwan Kim

**Affiliations:** Interdisciplinary Program for Bioengineering, Graduate School, Seoul National University, Seoul, 110-744 Korea; Interdisciplinary Program in Neuroscience, Graduate School, Seoul National University, Seoul, 151-742 Korea; Department of Rehabilitation Medicine, Seoul National University Hospital, Seoul, 110-744 Korea; Sensory Organ Research Institute, Seoul National University, Seoul, 151-742 Korea; Department of Neurosurgery, Seoul National University Hospital, Seoul, 110-744 Korea; Department of Brain and Cognitive Sciences, Seoul National University College of Natural Sciences, Seoul, 151-742 Korea; Department of Biomedical Engineering, Seoul National University College of Medicine, Seoul, 110-799 Korea; Institute of Medical and Biological Engineering, Seoul National University, Seoul, 151-742 Korea

**Keywords:** Brain–machine interface (BMI), Robot arm, Non-invasive, MEG, EEG, 3D trajectory

## Abstract

**Background:**

A brain-machine interface (BMI) should be able to help people with disabilities by replacing their lost motor functions. To replace lost functions, robot arms have been developed that are controlled by invasive neural signals. Although invasive neural signals have a high spatial resolution, non-invasive neural signals are valuable because they provide an interface without surgery. Thus, various researchers have developed robot arms driven by non-invasive neural signals. However, robot arm control based on the imagined trajectory of a human hand can be more intuitive for patients. In this study, therefore, an integrated robot arm-gripper system (IRAGS) that is driven by three-dimensional (3D) hand trajectories predicted from non-invasive neural signals was developed and verified.

**Methods:**

The IRAGS was developed by integrating a six-degree of freedom robot arm and adaptive robot gripper. The system was used to perform reaching and grasping motions for verification. The non-invasive neural signals, magnetoencephalography (MEG) and electroencephalography (EEG), were obtained to control the system. The 3D trajectories were predicted by multiple linear regressions. A target sphere was placed at the terminal point of the real trajectories, and the system was commanded to grasp the target at the terminal point of the predicted trajectories.

**Results:**

The average correlation coefficient between the predicted and real trajectories in the MEG case was $0.705 \pm 0.292$ (${\text{p}} < 0.001$). In the EEG case, it was $0.684 \pm 0.309$ (${\text{p}} < 0.001$). The success rates in grasping the target plastic sphere were 18.75 and 7.50 % with MEG and EEG, respectively. The success rates of touching the target were 52.50 and 58.75 % respectively.

**Conclusions:**

A robot arm driven by 3D trajectories predicted from non-invasive neural signals was implemented, and reaching and grasping motions were performed. In most cases, the robot closely approached the target, but the success rate was not very high because the non-invasive neural signal is less accurate. However the success rate could be sufficiently improved for practical applications by using additional sensors. Robot arm control based on hand trajectories predicted from EEG would allow for portability, and the performance with EEG was comparable to that with MEG.

## Background

In modern life, people face an elevated risk of losing all or part of their motor functions because of accidents or disease. Various technologies have been developed to help people who have lost their motor functions as replacements. A wearable robot arm driven by electromyography has been suggested [1], and functional electrical stimulation has been used to move the limb of a patient by stimulating a paralyzed muscle in lieu of inactive motor neurons [2]. This study used the brain-machine interface (BMI), which allows the neural signals of the user to be analyzed to realize the control of external machines. In other words, BMI allows a person to bypass conventional neuromuscular pathways to interact with the environment [3].

Since the BMI concept was first proposed in the 1970s at the University of California Los Angeles [4, 5], scientists and engineers have improved upon the technology to develop human-controlled external effectors that do not require physical movement [6–9]. Recently, monkeys have fed themselves by controlling a robotic arm [10], and humans have used invasive neural signals to control a seven-degree of freedom (DOF) robot arm as if it were their own arm [11, 12]. An invasive electrode array collects the neural signal directly from the brain tissues. Therefore, it has the advantage of high spatial resolution, which allows for accurate prediction of the human intention. In these studies, robot arms were controlled according to the predicted three-dimensional (3D) trajectories of a human hand. The velocity vector of the human hand was predicted from neural signals. This approach enables subjects to control a robot arm intuitively as if it were their own arm.

**Fig. 1:**
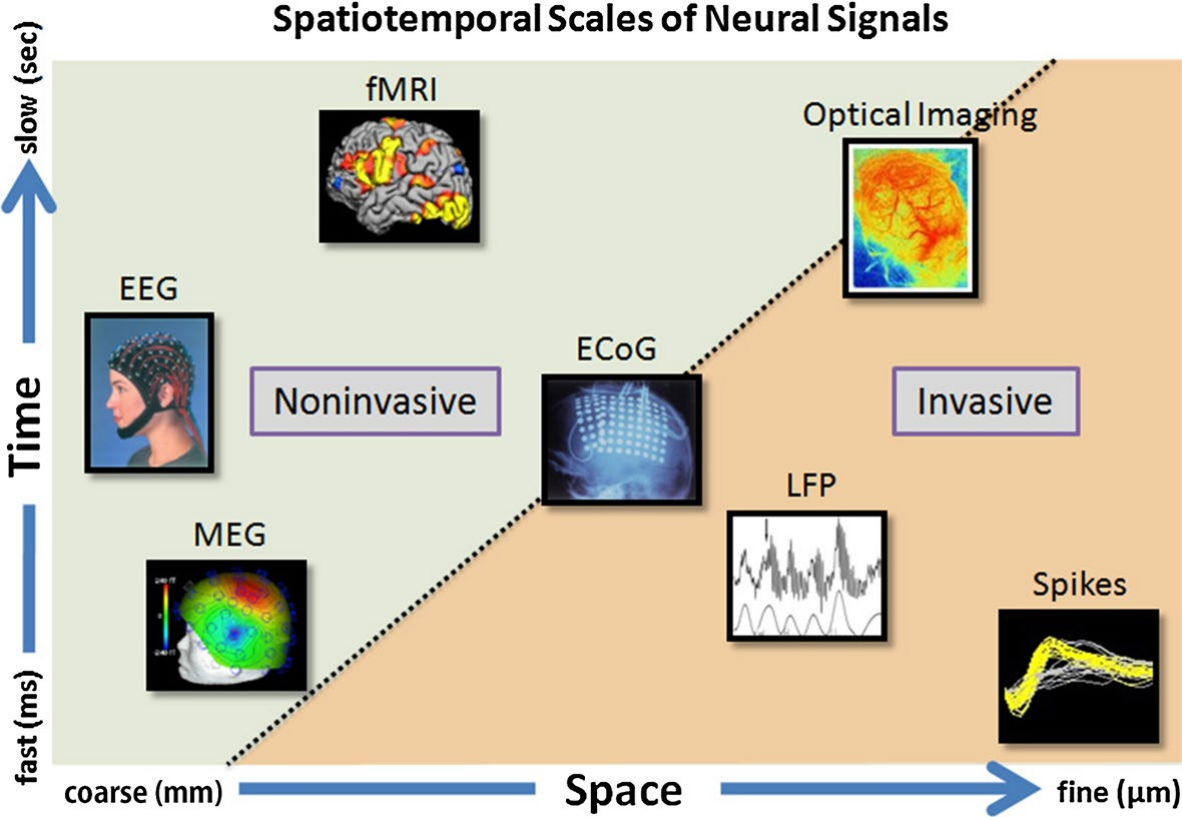
Spatiotemporal scale of neural signals. Spatiotemporal scales of neural signals that can potentially be used for brain–machine interfaces. Invasive neural signals have a fine spatial resolution, whereas non-invasive neural signals such as EEG and MEG have a coarse spatial resolution. ECoG has a spatial resolution between the other two

Researches working on robot arm control based on predicting the hand trajectory have considered using not only invasive neural signals but also less-invasive neural signals such as electrocorticography (ECoG). Because the electrodes of ECoG contact the surface of the brain tissue, it provides a spatial resolution between that of invasive and non-invasive neural signals (see Fig. 1; [13]). A mesh of ECoG electrodes is inserted beneath the skull and draped over the surface of the arachnoid. Recently, ECoG has been used to predict hand movement trajectories and classify grasping types [14–19].

Even though invasive and less-invasive neural signals demonstrate a relatively high spatial resolution, non-invasive neural signals such as electroencephalography (EEG) and magnetoencephalography (MEG) are valuable because they provide an interface without surgery. In particular, EEG can provide a practical interface because of its portability. Although the safety of surgery for electrode implantation has been confirmed by researchers, it is still burdensome for patients. Furthermore, the surgery for electrode implantation carries the risk of causing brain infection, bleeding, and brain tissue damage [9]. Therefore, researches have considered robot arm control based on non-invasive neural signals. Valbuena et al. [20] and Bakardjian et al. [21] used steady-state visual evoked potential (SSVEP) to control a seven-DOF robot arm. Pathirage et al. [22] used P300 to control a wheelchair-mounted robot arm. Some research groups succeeded at moving a target object by using a multi-DOF robot arm or wearable robot suit [23–25]. Valenzuela and Avila [26] proposed biomimetic control of mechanical systems equipped with musculotendon actuators, which can potentially be activated by non-invasive neural signals. These studies have contributed to increasing the accuracy of robot arm control based on using non-invasive neural signals. However, predicting the hand trajectory can provide more intuitive control for patients than previous methods because it allows the robot arm end-effector to be controlled how patients imagine moving their own arm. It provides the possibility of a damaged human arm being completely replaced by a robotic arm that moves as if it were the user’s own arm. Thus, various researchers have studied the use of non-invasive neural signals to predict hand trajectories. Recently, 3D trajectories have been predicted by using human EEG and MEG [27–31], which demonstrates the potential for more intuitive robot arm control. Bradberry et al. [31] reported results for the reconstruction of 3D movement trajectories by using EEG. However, the accuracy was not high enough (correlation coefficient = 0.19–0.38). A plausible explanation for the low accuracy is that the EEG was low-pass filtered at 1 Hz, even though movement-evoked potentials during arm movements include components that are faster than 1 Hz [32]. Other groups have tried to improve the accuracy by changing the filtering frequency (0.5–8 Hz) and methods [27–30]. Not only the frequency band, but also other two factors were additionally changed to improve the performance. The studies have provided sufficient rest time (4 s) between reaching behaviors to exclude mere fluctuations from analysis. Furthermore, the study used 200 ms interval data (from −200 ms to present) whereas previous EEG study used 100 ms interval data. Through these changes, the accuracy was improved (correlation coefficient > 0.7) compared to that of the EEG study. However, the new methods have not yet been applied with EEG, which would allow for a portable BMI system.

Reports on robot arms controlled by 3D hand trajectories predicted from non-invasive neural signals are scarce. Even though robot arm control based on 3D hand trajectories is a challenging topic, it can contribute to the implementation of a more intuitive BMI system. In the present study, an integrated robot arm-gripper system (IRAGS), that is driven by 3D hand trajectories predicted from MEG and EEG was developed. The improved method proposed in recent studies [27–30] is used to obtain accurate trajectory. This is the first study on controlling a robot arm by using 3D hand trajectories predicted from non-invasive neural signals. The performance of the system was verified by the performance of reaching and grasping motions, which are important components of the BMI system [33]. The Performance with MEG and EEG were compared to consider the portability of the proposed system.

## Methods

### System overview

**Fig. 2:**
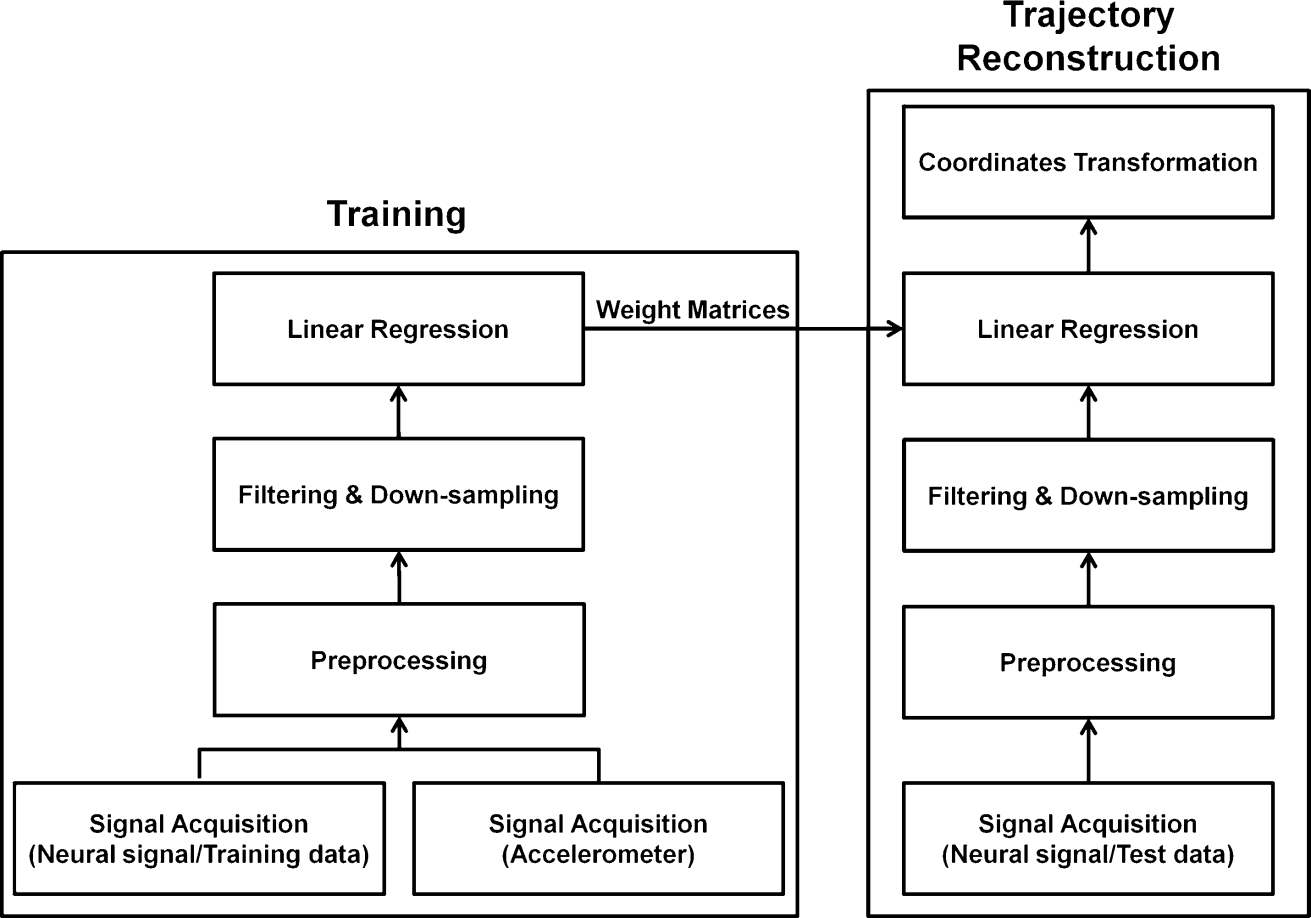
Signal processing procedure. Training has to be conducted to obtain the weight matrices. The filtering, down-sampling, and linear regression processes are explained in the “Movement prediction” section

The BMI system contains two major subsystems: the signal processing system and IRAGS. The signal processing system consists of signal acquisition, preprocessing, movement prediction, and coordinate transformation. Figure 2 shows the signal processing procedure, which is explained in the next section. The IRAGS is a robot arm consisting of two industrial robots: a six-DOF robot arm (VS-6556G, DENSO, Kariya, Aichi Prefecture, Japan) and an adaptive robot gripper (three-finger adaptive gripper, Robotiq, Saint-Nicolas, QC, Canada).

In the following subsections, the signal processing system and IRAGS are introduced first. Then, the process of offline verification is explained.

### Signal processing system

#### MEG signal acquisition and preprocessing

The acquisition of neural signals and the processing procedures are the same as those described in a previous study [27]. MEG signals are acquired from 306 channels of a whole-head MEG system (VectorView TM, Elekta Neuromag Oy, Helsinki, Finland) in a magnetically shielded room. The 306 channels consist of 204 planar gradiometers and 102 magnetometers distributed at 102 locations. The sampling frequency is 600.615 Hz, and the signal is band-pass filtered in the range of 0.1–200 Hz. To eliminate external noise, the spatiotemporal signal space separation (tSSS) method is used. The neural signals are segmented from −1 s before the cue onset to 2 s after the cue onset and band-pass filtered in the range of 0.1–100 Hz. The 68 gradiometers of the 306 channels in the motor-related area are selected for movement prediction. The 68 gradiometers include motor-related areas [34] and demonstrate event-related desynchronization (ERD) around the alpha (8–13 Hz) and beta (13–30 Hz) frequencies [35]. An accelerometer (KXM52, Kionix, NY, USA) is placed on the index finger, and the sensor signals are simultaneously recorded with MEG at the same sampling rate.

#### EEG signal acquisition and preprocessing

EEG signals are measured by using a 64-channel EEG system (Synamps 2, Compumedics Neuroscan, Texas, USA). The sampling frequency is 1000 Hz and low-pass filtered at 200 Hz. A notch filter is applied at 60 Hz to remove line noise. The signals are segmented from -1 s before the cue onset to 2 s after the cue onset. All 64 channels are used for movement prediction. Because the number of EEG channels is insufficient, in contrast to MEG, all channels are used to maximize accuracy even though they are distributed in not only the motor-related areas but also other areas. The accelerometer signals are simultaneously acquired with the EEG signals at the same sampling frequency.

#### Movement prediction (filtering, down-sampling, and linear regression)

The MEG and EEG signals are band-pass filtered in the range of 0.5–8 Hz. The accelerometer signals are filtered in the range of 0.2–5 Hz. The movement velocity is calculated by integrating the accelerometer signals with respect to time. The filtered neural signals are down-sampled at 50 Hz (20 ms intervals). The movement velocities are also down-sampled at 50 Hz (20 ms intervals). Neural signals with 200 ms intervals (average of one current point and 10 preceding points) are used as features to predict the present velocity. The *x*, *y*, and *z* velocities of the movements are predicted from the neural signal by using multiple linear regression. The regression equations are expressed below in Eqs. 1, 2, 3.

1 $$V_{x} \left( {\text{t}} \right) = \mathop \sum \limits_{i = 1}^{n} \mathop \sum \limits_{j = 0}^{m} W_{ij}^{x} \times S_{i} \left( {t - j} \right) + W_{0}^{x}$$

2 $$V_{y} \left( {\text{t}} \right) = \mathop \sum \limits_{i = 1}^{n} \mathop \sum \limits_{j = 0}^{m} W_{ij}^{y} \times S_{i} \left( {t - j} \right) + W_{0}^{y}$$

3 $$V_{z} \left( {\text{t}} \right) = \mathop \sum \limits_{i = 1}^{n} \mathop \sum \limits_{j = 0}^{m} W_{ij}^{z} \times S_{i} \left( {t - j} \right) + W_{0}^{z}$$

$V_{x} \left( {\text{t}} \right)$, $V_{y} \left( {\text{t}} \right)$, and $V_{z} \left( {\text{t}} \right)$ are the calculated velocities from the accelerometer. $W_{ij}^{x}$, $W_{ij}^{y}$, and $W_{ij}^{z}$ are the weight matrices, and $S_{i}$ is the MEG/EEG signal of the *i*th channel. *n* is the number of channels (68 for MEG and 64 for EEG), and *m* is the number of data points before the time *t*. The weight matrices are obtained first by training. Then, the weight matrices are used to predict the velocities from the neural signals. The trajectories are calculated by integrating the predicted velocities as given below in Eqs. 4 and 5.

4 $$\overrightarrow {V(t)} = [V_{x} (t) V_{y} (t) V_{z} (t)]$$

5 $$\overrightarrow {P(\tau )} = \mathop \smallint \nolimits_{t = 0}^{\tau } \overrightarrow {V(t)} dt$$

$\overrightarrow {V(t)}$ is the predicted velocity vector at the time *t*, and $\overrightarrow {P(\tau )}$ is the position vector at the time $\tau$.

#### Coordinate transformation

**Fig. 3:**
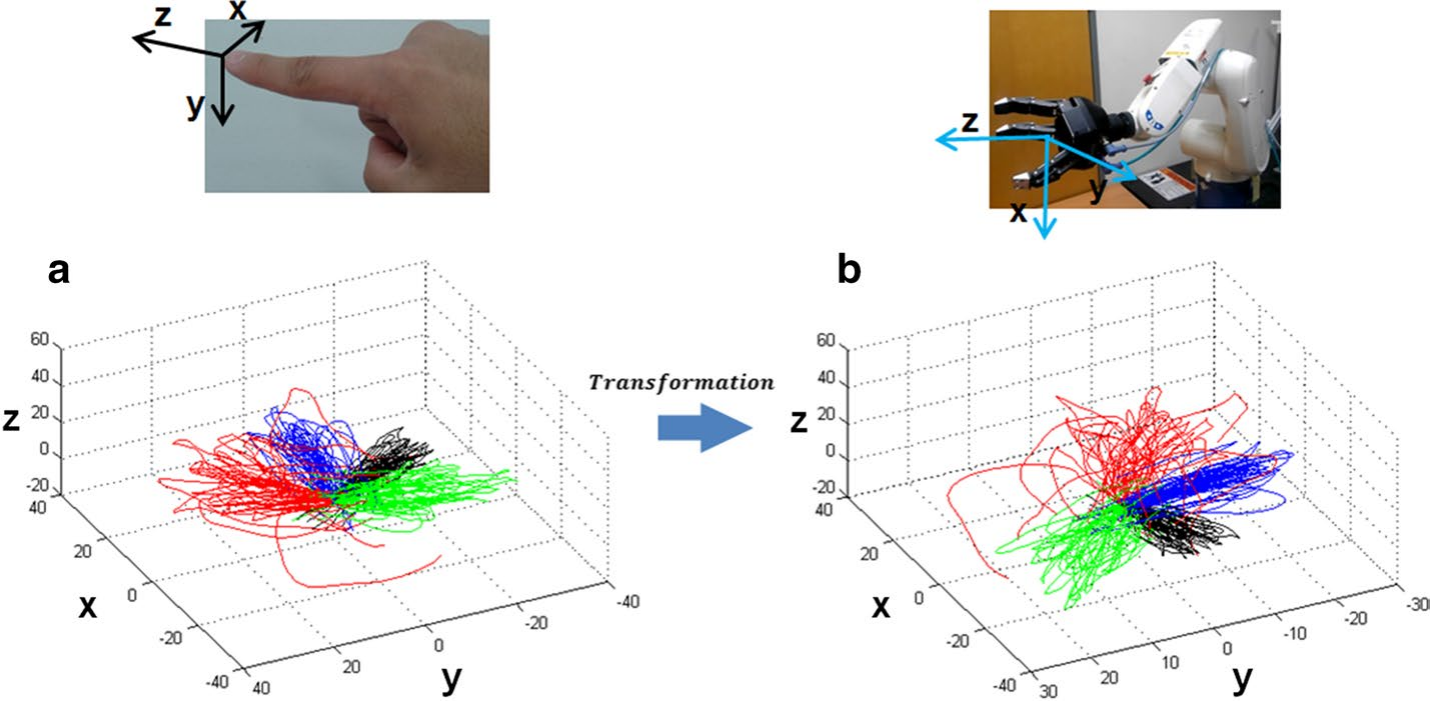
Rotational transformation to change trajectories in the accelerometer coordinates to IRAGS coordinates. **a** Predicted hand trajectories reconstructed from neural signal before transformation. **b** Predicted hand trajectories after transformation. *IRAGS* integrated robot arm-gripper system

The 3D coordinates defined for the accelerometer and IRAGS are different. Therefore, they should be represented in one form to be controlled by a single system. The coordinates of the IRAGS is defined as the reference frame, and the coordinates of the accelerometer is transformed into the IRAGS coordinates. We assumed that the index finger is maintained at an angle of up to 30° from the horizontal plane. To maintain the angle between the index finger and horizontal plane at a constant value, the subjects were instructed to maintain their finger at the initial orientation. Although the invasive BMI studies achieved control of a robot arm with seven DOFs (three for translational movements, three for orientation, and one for grasping) [11, 12], the present study focused only on the three DOFs of the translational movements. Therefore, the orientation of the index finger should be fixed to acquire the signal, which is not affected by the orientation movement of the hand. An additional method of controlling the orientation in future study is discussed in the Discussion section. Coordinate transformation is performed by multiplying the rotational matrices. The rotation axis is defined as matrix *A* in Eq. 6, and the rotation matrix is represented as $R_{A} (\theta )$ in Eq. 7, where $\theta$ is the rotated angle. By multiplying the two rotation matrices as expressed in Eq. 8, the trajectory can be transformed from the accelerometer coordinates to the IRAGS coordinates. Figure 3 shows the coordinates of each system and the trajectories before and after the transformation as an example.

6 $$A = \left[ {\begin{array}{*{20}c} {A_{1} } & {A_{2} } & {A_{3} } \\ \end{array} } \right]$$

7 $$R_{A} \left( \theta \right) = \left[ {\begin{array}{*{20}c} {cos\theta + (1 - cos\theta )A_{1}^{2} } & {\left( {1 - cos\theta } \right)A_{1} A_{2} - sin\theta A_{3} } & {\left( {1 - cos\theta } \right)A_{1} A_{3} + sin\theta A_{2} } \\ {\left( {1 - cos\theta } \right)A_{1} A_{2} + sin\theta A_{3} } & {cos\theta + (1 - cos\theta )A_{2}^{2} } & {\left( {1 - cos\theta } \right)A_{2} A_{3} - sin\theta A_{1} } \\ {\left( {1 - cos\theta } \right)A_{1} A_{3} - sin\theta A_{2} } & {\left( {1 - cos\theta } \right)A_{2} A_{3} + sin\theta A_{1} } & {cos\theta + (1 - cos\theta )A_{3}^{2} } \\ \end{array} } \right]$$

8 $$Coord_{robot} = R_{y} ( - 30^\circ ) \cdot R_{z} ( - 90^\circ ) \cdot Coord_{accelerometer}$$

### IRAGS

#### Six-DOF robot arm

**Fig. 4:**
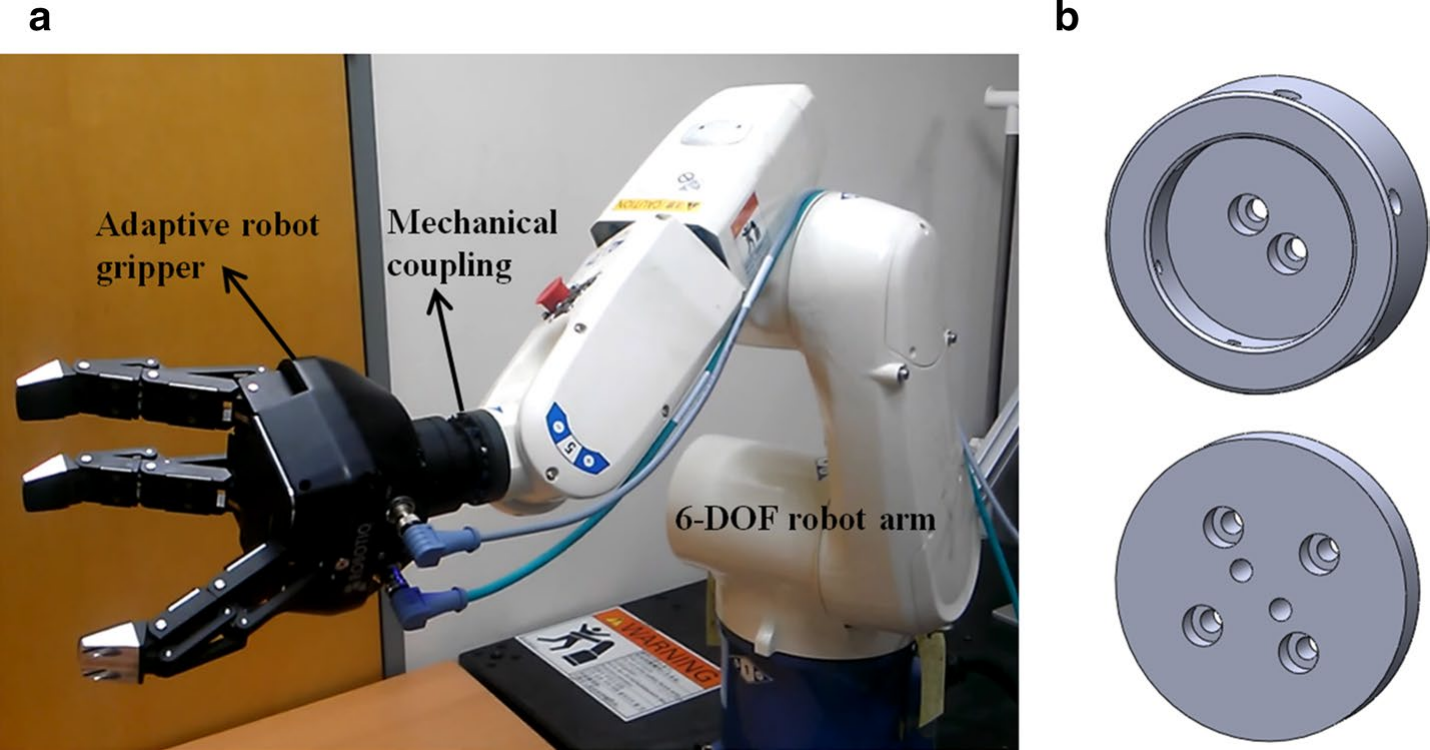
Integrated robot arm-gripper system (IRAGS). **a** Hardware of the IRAGS. The IRAGS consists of a six-DOF robot arm, adaptive robot gripper, and mechanical coupling. **b** Mechanical coupling to connect the six-DOF robot arm and adaptive robot gripper. *IRAGS* integrated robot arm-gripper system

A six-DOF robot arm movement consists of a translational movement (three DOFs) and orientation rotation (three DOFs). The translational movement is predicted by neural signals, whereas the orientation rotation is assigned to maintain the end-effector in the horizontal direction. Therefore, the six-DOF robot arm is controlled with three DOFs. In a recent study, Bennis and Roby-Brami found that the orientation of the human hand is closely related to its velocity vector [36]. However, the orientation is not that significant when the object has a spherical shape, and a spherically shaped object was used for grasping in this study. The robot arm is controlled by an algorithm based on Microsoft Visual Studio 2010 (Microsoft, Redmond, WA, USA). The six-DOF robot arm contains an external controller, and it communicates by using a personal computer through a binary controller access protocol. The six-DOF robot arm is shown in Fig. 4a.

#### Adaptive robot gripper

The industrial adaptive gripper is an optimized machine for grasping. The adaptive robot gripper consists of three fingers and has five DOFs. Three of the five DOFs are used for the grasping motion, and the others are used for the lateral motion of two fingers. The five DOFs are coupled to a single-DOF motion to grasp a spherical object based upon a simple command. A controller is installed inside the robot. The transmission control protocol and internet protocol are used for communication. The control algorithm is implemented with MATLAB R2013b (MathWorks, Natick, MA, USA). The maximum grasping force and speed are set to 15 N and 22 mm/s, respectively. The internal controller stops the grasping motion when each finger reaches the assigned maximum grasping force. Power is supplied from a regulated direct-current power supply (PWS-3005D, Provice, Hwaseong-si, Gyeonggi-do, Korea) and the voltage is set to 24 V. The adaptive robot gripper is shown in Fig. 4a.

#### Robot system integration

To integrate the robot arm and the gripper, a mechanical coupling was designed. The implemented IRAGS and mechanical coupling are shown in Fig. 4a, b, respectively. The robot arm is controlled by an algorithm based on Microsoft Visual Studio, and the adaptive robot gripper is controlled by an algorithm based on MATLAB. To control the IRAGS with a single algorithm, the system is implemented so that the robot arm and robot gripper can interact. If a grasping signal is provided to the IRAGS, the adaptive robot gripper performs a grasping motion, while the robot arm stops moving. The IRAGS is controlled with four DOFs. Three DOFs are for the translational movement of the six-DOF robot arm, and one DOF is for the grasping motion of the adaptive robot gripper.

### Offline verification

To verify the stability and performance of the IRAGS, it was used to perform the reaching and grasping of a target object. The success rate of the reaching and grasping motions with the IRAGS was measured to predict whether the system can be used in real-world situations. As a preliminary study, the input (neural signal) was provided offline.

**Fig. 5:**
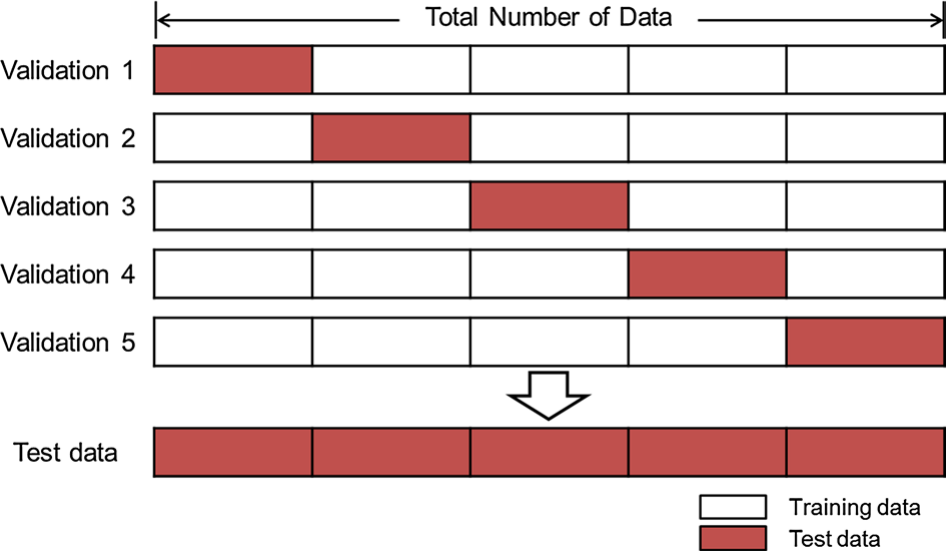
Fivefold cross validation for hand trajectory prediction. In the fivefold cross validation process, the test data, which guarantee generalizability, is obtained

As a first step, MEG and EEG signals were acquired, and the trajectories of the human arm movement were predicted. The neural activity was measured during the reaching movements with MEG and EEG. Nine healthy subjects for each signal (MEG case: 19–37 years old, five males and four females; EEG case: 25–31 years old, five males and four females) participated in the study. Stereographic images were presented to the subjects to guide the reaching movements. At the start of the experiment, an image of a sphere was presented at the center of a screen, and each subject was instructed to put his/her index finger on the sphere. After 4 s, a target sphere appeared randomly at each of the four corners of the screen. The subject was instructed to move the index finger to the target sphere and then move it back to the center. These reaching movements were repeated during the experiments. Two sessions were performed by each subject. For each session, the subjects performed reaching movements for 30 trials in each direction. The experiment was approved by the Institutional Review Board (IRB) of Seoul National University Hospital (IRB No.: 1501-006-637). The trajectories were predicted by fivefold cross validation. This method separates four-fifths of the data for training (obtaining weight matrices) and one-fifth for testing. Thus, five combinations of training and testing data were available. Through the validation, test data was obtained. The method of cross validation has been used in previous studies to obtain generalizability [27, 31]. The process of fivefold cross validation for predicting 3D hand trajectory is demonstrated in Fig. 5. The length of the trajectories reconstructed by integrating the accelerometer signal was scaled to 30 cm by multiplying the scaling coefficient. The scaling coefficient was used to scale the reconstructed trajectories derived from the neural signal.

As a second step, the position of the target object was defined. A plastic sphere was fixed at a position as a target. To define the position of the target object, information from a real-limb trajectory was used. Even though no real plastic sphere existed when the neural signal was acquired, the subjects felt their fingers reached the target object (the stereographic images) when their arms were completely stretched. Therefore, we fixed the target object at the average of the terminal positions of the real trajectories. By using the accelerometer data, the average of the real terminal positions in each direction (four directions) and in each session (18 sessions) was calculated. The values of the *x*, *y*, and *z* coordinates were averaged, as expressed in Eqs. 9, 10, 11.

9 $$x_{avg} = \frac{1}{30}\mathop \sum \limits_{i = 1}^{30} x_{term, i}$$

10 $$y_{avg} = \frac{1}{30}\mathop \sum \limits_{i = 1}^{30} y_{term, i}$$

11 $$z_{avg} = \frac{1}{30}\mathop \sum \limits_{i = 1}^{30} z_{term, i}$$

[x_avg_ y_avg_ z_avg_] is the calculated position to fix the target object, and [x_term,i_ y_term,i_ z_term,i_] is the terminal position of the *i*th trajectory in each session. The diameter of the target sphere was 70 mm, which is approximately the size of a baseball (a baseball has a circumference of approximately 23 cm, for a diameter of approximately 73 mm) and is a comfortable size for grasping by an average person.

As a final step, IRAGS was used to perform reaching and grasping motions. To dexterously perform reaching and grasping motions, seven DOFs are necessary (three for translational movement, three for orientation, and one for grasping) [37]. Only the three DOFs of the translational movement were predicted in the present study, and the three DOFs of the orientation were fixed as constant. A pseudo-grasping signal was provided for the grasping motion. The pseudo-grasping signal was automatically provided when the distance between the adaptive robot gripper and target sphere was at its shortest.

The accuracy of the predicted trajectories was evaluated by calculating the correlation, root mean square error (RMSE) and terminal point error (TPE). The grasping and touching target success rates were also measured.

## Results

### Predicted trajectory

#### Accuracy of the predicted trajectory

**Fig. 6:**
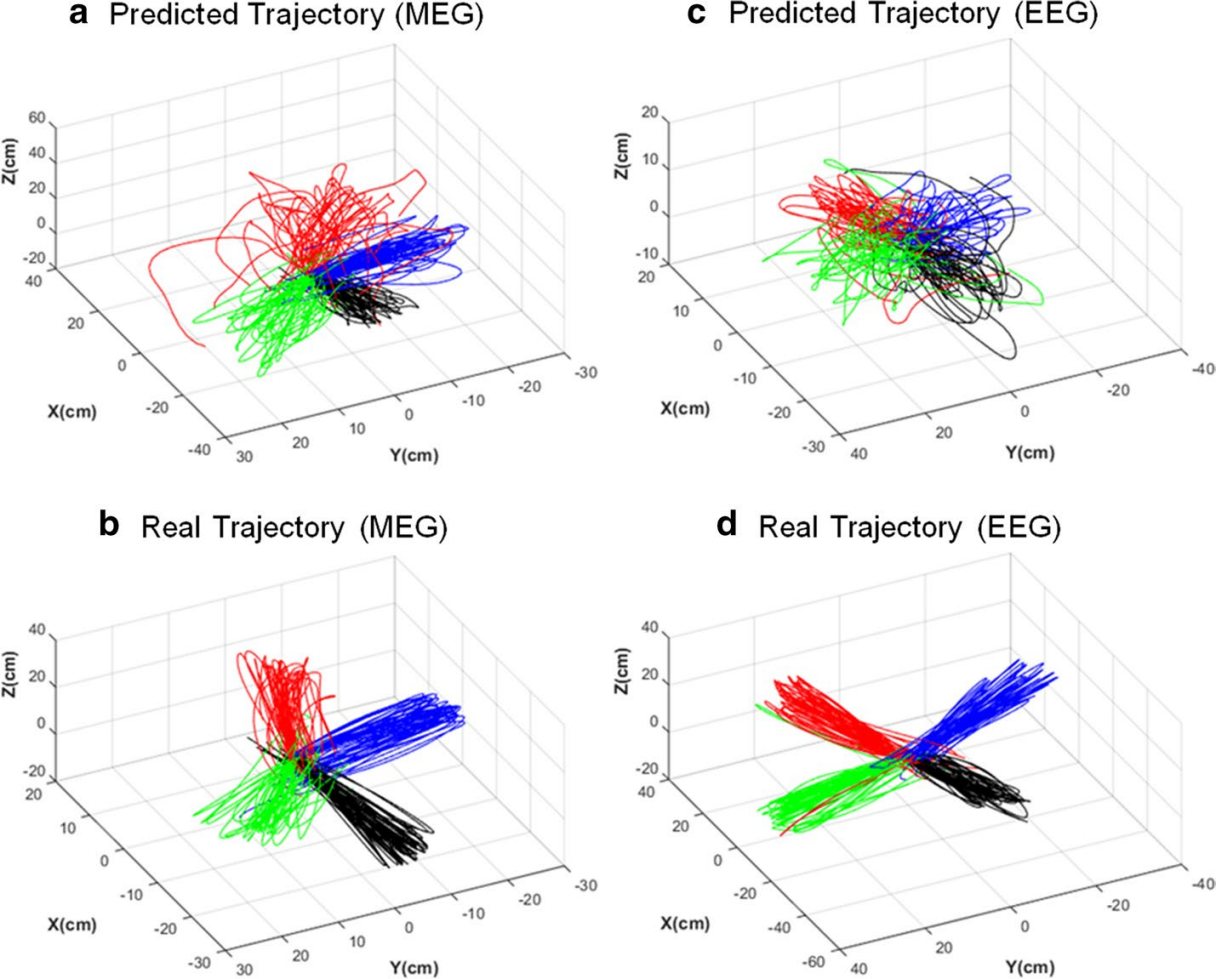
Trajectories predicted from neural signals and real trajectories. **a** Hand trajectories predicted by MEG (first session of subject 4). **b** Real hand trajectories reconstructed from the accelerometer signal during MEG signal acquisition (first session of subject 4) **c** Hand trajectories predicted by EEG (first session of subject 7). **d** Real hand trajectories reconstructed from the accelerometer signal during EEG signal acquisition (first session of subject 7). *Each color* represents one of the four hand-movement directions

**Fig. 7:**
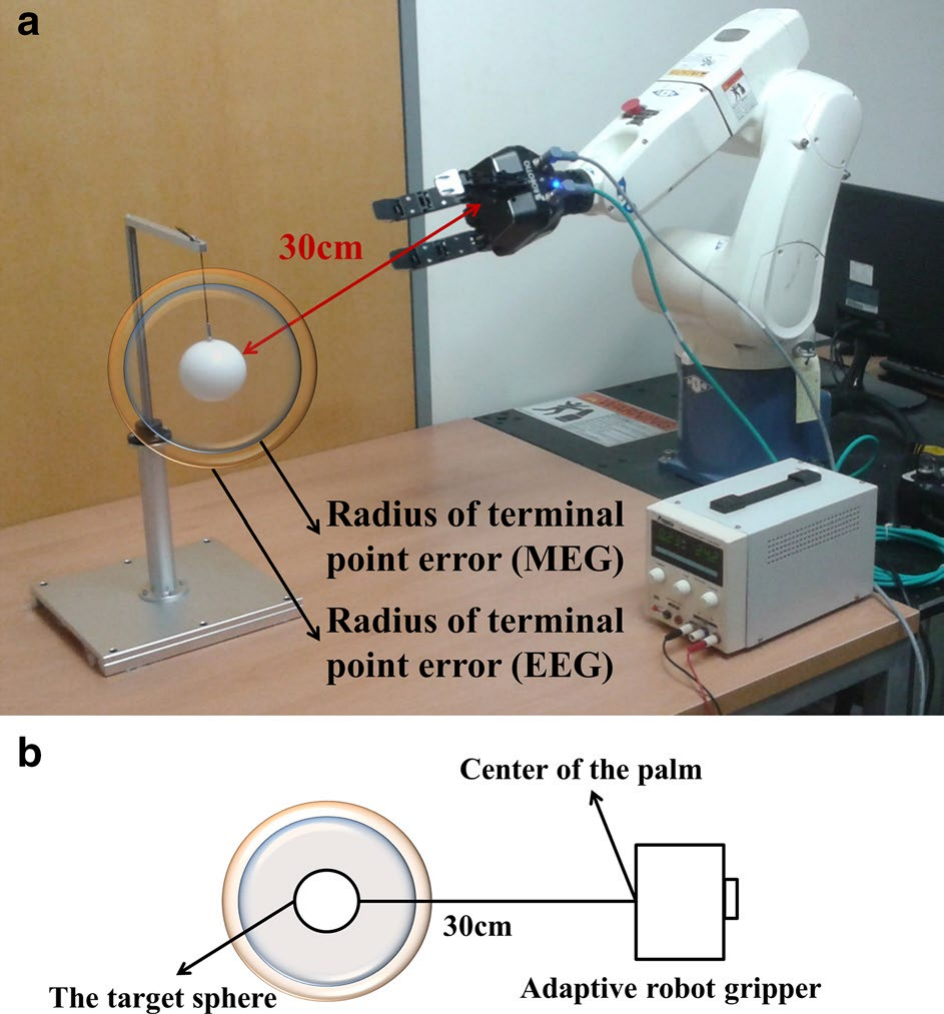
TPE of MEG and EEG. **a** The distance between the target sphere and adaptive robot gripper was 30 cm. The radius of the TPE is the average distance between the target sphere and adaptive robot gripper at the closest position. **b** Miniaturized drawing of the real BMI system. Lengths are proportional to the actual size. *TPE* terminal point error

MEG was measured for two sessions from each of the nine subjects. Each session consisted of 120 trials. In total, 2,160 trials were conducted with MEG. As an example, Fig. 6a shows the predicted trajectories from the first session of subject 4. Figure 6b shows the real trajectories derived from the accelerometer. The correlation coefficient, RMSE, and TPE were measured to evaluate the accuracies of the predicted trajectories. These are listed in Table 1. The average correlation coefficients from each session were significant (${\text{p}} < 0.0027$ for the least accurate session). The total correlation coefficient between the real and predicted trajectories was $0.705 \pm 0.292$ (${\text{p}} < 0.001$) on average. The data from subject 2 exhibited a low correlation coefficient because the reaching behavior of the subject during the task was inconsistent. RMSE is an index that indicates the average Euclidean distance between the real and predicted trajectories. The average RMSE was $11.154 \pm 5.399 {\text{cm}}$. Except for three sessions (one session contained an outlier, and the other two sessions were from subject 2 who exhibited inconsistent reaching behavior was performed), all other sessions exhibited an RMSE of less than 12 cm. TPE indicates the distance between the surface of the target sphere and the predicted trajectory at the closest position. The pseudo-grasping signal was provided at the closest position. Therefore, TPE is an index that is closely related to the success rate of grasping the target. The average TPE was $9.714 \pm 4.789 {\text{cm}}$. Figure 7a, b show the BMI system to provide a reference for the readers with regard to the degree of accuracy. Figure 7b shows a miniaturized drawing of a real BMI system, although the palm of the adaptive robot gripper is simplified as squares. The radius of the smaller transparent sphere around the target represents the TPE of the MEG. This implies that, on average, the center of the palm of the adaptive robot gripper approached the surface of the transparent sphere.

**Table 1:** Accuracy of the trajectories predicted with MEG

Subject	Session	Correlation	RMSE (cm)	TPE (cm)
1	1	0.726 (0.224)	10.041 (3.584)	11.040 (4.334)
	2	0.706 (0.216)	10.165 (3.458)	11.068 (4.490)
2	1	0.513 (0.381)	17.418 (7.194)	16.086 (6.086)
	2	0.569 (0.321)	16.953 (9.258)	4.027 (2.205)
3	1	0.812 (0.166)	13.383 (6.544)	6.499 (4.175)
	2	0.820 (0.188)	20.006 (70.367)	7.595 (4.786)
4	1	0.762 (0.219)	7.935 (3.147)	8.239 (3.241)
	2	0.800 (0.233)	6.919 (3.108)	6.787 (3.537)
5	1	0.754 (0.210)	8.560 (3.080)	8.086 (3.483)
	2	0.657 (0.269)	11.038 (4.098)	10.351 (4.849)
6	1	0.654 (0.265)	8.401 (3.299)	9.004 (4.372)
	2	0.770 (0.233)	6.811 (1.880)	8.391 (3.118)
7	1	0.728 (0.196)	10.304 (3.656)	10.564 (4.397)
	2	0.750 (0.210)	8.745 (3.098)	8.395 (4.076)
8	1	0.699 (0.274)	11.379 (6.137)	12.190 (5.771)
	2	0.762 (0.209)	10.522 (8.859)	11.621 (4.163)
9	1	0.620 (0.277)	11.022 (4.401)	11.346 (6.023)
	2	0.584 (0.246)	11.169 (3.924)	12.850 (6.033)
Average		0.705 (0.292)	11.154 (5.399)	9.714 (4.789)

Values in brackets represent standard deviations

*RMSE* root mean square error, *TPE* terminal point error

Then, 2160 trials were conducted with EEG. As an example, Fig. 6c shows the predicted trajectories. Figure 6d shows the real trajectories derived from the accelerometer. The same indexes were measured to evaluate the trajectory accuracy. These are listed in Table 2. The average correlation coefficients from each session were significant (p < 0.011 for the least accurate session). Further, the total correlation coefficient between the real and predicted trajectories was $0.684 \pm 0.309$ (${\text{p}} < 0.001$) on average, and the RMSE was $13.724 \pm 5.370$ cm on average. The TPE was $11.432 \pm 4.749$ cm on average. The radius of the larger transparent sphere shown in Fig. 7 represents the TPE of the EEG.

**Table 2:** Accuracy of the trajectories predicted with EEG

Subject	Session	Correlation	RMSE (cm)	TPE (cm)
1	1	0.777 (0.204)	19.606 (58.826)	4.539 (2.771)
	2	0.745 (0.209)	11.569 (13.751)	11.915 (4.293)
2	1	0.743 (0.333)	14.908 (8.867)	12.007 (4.061)
	2	0.592 (0.255)	19.262 (37.090)	14.923 (4.700)
3	1	0.743 (0.202)	12.694 (17.501)	10.547 (4.033)
	2	0.756 (0.224)	11.065 (6.688)	9.923 (3.766)
4	1	0.587 (0.197)	15.007 (3.204)	13.643 (4.747)
	2	0.729 (0.205)	12.267 (2.833)	9.369 (3.005)
5	1	0.437 (0.459)	17.402 (10.729)	7.726 (5.526)
	2	0.439 (0.539)	17.590 (7.753)	8.854 (7.744)
6	1	0.635 (0.271)	14.435 (3.482)	8.486 (5.152)
	2	0.569 (0.308)	15.037 (3.454)	10.115 (4.403)
7	1	0.592 (0.277)	12.385 (3.259)	17.309 (4.899)
	2	0.820 (0.175)	11.011 (2.415)	13.828 (3.380)
8	1	0.787 (0.186)	12.225 (3.719)	14.689 (3.600)
	2	0.798 (0.129)	10.773 (3.181)	12.563 (3.875)
9	1	0.800 (0.155)	10.158 (2.485)	12.852 (4.224)
	2	0.765 (0.198)	9.638 (2.846)	12.482 (4.333)
Average		0.684 (0.309)	13.724 (5.370)	11.432 (4.749)

Values in brackets represent standard deviations

*RMSE* root mean square error, *TPE* terminal point error

#### Comparison of MEG and EEG results

The trajectories predicted with MEG and EEG both had a correlation coefficient of approximately 0.7. This implies that the predicted trajectories were significantly correlated to the real trajectories (because p < 0.03 for all sessions). According to the statistical analysis, EEG provided significantly less accuracy than MEG. Using an independent-sample comparison, we compared the three indexes. All three indexes with MEG were significantly (p < 0.03) better than those with EEG (see Tables 1, 2). These results are widely known in the BMI field [13]. However, the practical values exhibited a small difference. The RMSE exhibited a difference of 2.566 cm, and the TPE exhibited a difference of only 1.717 cm. Figure 7b shows that the two transparent spheres had a small size difference. The effect sizes listed in Table 3 (Cohen’s d < 0.50) also demonstrate that the difference between EEG and MEG was not critical despite the proven statistical significance.

**Table 3:** Comparison of MEG and EEG

	Correlation	RMSE	TPE
p value	0.011	<0.001	<0.001
Cohen’s d	0.070	0.477	0.360

In generally, the effect size is not critical when Cohen’s d is less than 0.5 [38]

### Performance of reaching and grasping motions

Before the reaching and grasping motions were performed, the accuracy of the IRAGS was verified in an environment using an approximately 2.3 kg robot gripper fixed on the end-effector. Twelve trials (three trials per direction) were randomly selected among the predicted trajectories from the MEG and were used to verify the accuracy of the robot system. The RMSE between the input trajectory (predicted trajectory) and robot trajectory was $1.8 \times 10^{ - 3} \pm 3.3 \times 10^{ - 3} {\text{cm}}$. This is an extremely small error, and no further calibration was needed.

**Fig. 8:**
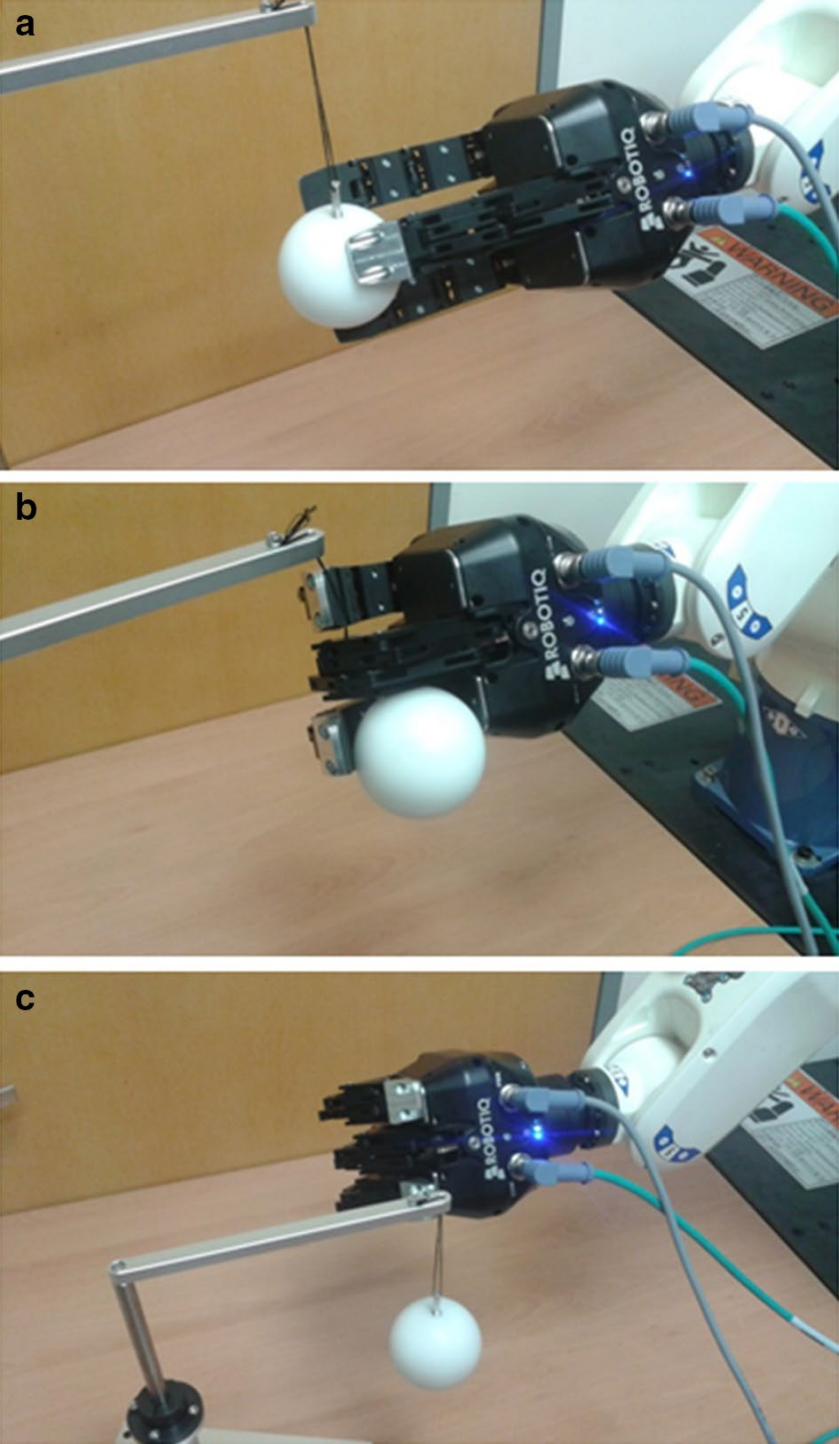
Grasping and touching the target object. **a** Grasping. **b** Touching. **c** Failure

To verify the reaching and grasping motions, 80 trials from two sessions that demonstrated the median correlation coefficient were selected (40 trials from each session) as input data. To test the reliability of the experiment, trials using the median correlation coefficient were selected as representative. The experiment was conducted with both EEG and MEG. Forty trials were randomly selected from the first session of subject 1 and the first session of subject 7 for MEG. For EEG, 40 trials were randomly selected from the first session of subject 2 and the first session of subject 3. The results were classified into three types: grasping, touching, and failure. These are shown in Fig. 8. A grasping success was counted as both grasping and touching. Out of the 80 trials, 15 trials (18.75 %) with MEG and six trials (7.50 %) with EEG succeeded at grasping, as listed in Table 4. However, the success rate was much higher for touching the target than grasping it. With MEG, 42 out of 80 trials (52.50 %) succeeded in touching the target. With EEG, 47 out of 80 trials (58.75 %) succeeded in touching the target. The touching success rates were similar between MEG and EEG, whereas the grasping success rate was clearly higher in the MEG case.

**Table 4:** Number of successes and success rates for grasping and touching

Signal type	Grasping		Touching	
	No. of successes	Success rate (%)	No. of successes	Success rate (%)
MEG	15	18.75	42	52.50
EEG	6	7.50	47	58.75

MEG and EEG were each used in 80 trials

## Discussion

Intuitive robot arm control based on non-invasive neural signals is preferred for potential patients who have lost their motor functions. In this study, the non-invasive neural signals, EEG and MEG, were considered to meet this objective. The possibility of a portable BMI system for robot arm control was also considered by comparing the accuracies of EEG and MEG.

### Evaluation for the accuracy

Both EEG and MEG exhibited a high correlation between the predicted and real trajectories. In particular, EEG showed improved accuracy compared to the results of a previous EEG study [31]. In our study, three factors were mainly improved. First, frequency band was changed to the broader pass band (0.5–8 Hz) to cover movement information of higher frequency. Furthermore, sufficient rest time (4 s) was provided to exclude mere fluctuations from analysis. As a final factor, the study used 200 ms interval data (from −200 ms to present) whereas previous EEG study used 100 ms interval data. The previous MEG study [27] showed that correlation coefficient increases as the interval of preceding data increases. The three factors, which were already applied to MEG study [27], contributed to performance improvement not only for the MEG case but also EEG case.

Even though the trajectories were relatively accurate, they were not sufficient for performing reaching and grasping motions by IRAGS. Three issues could be proposed as the reasons for the low accuracy. The first is the limited spatial resolution of the non-invasive neural signals. Non-invasive neural signals are known to have a coarse spatial resolution, as shown in Fig. 1, because the electrodes are located on the surface of the human head. In contrast, the invasive neural sensors are directly implanted into the tissue of the human brain.

The second factor is the inaccuracy of the accelerometer. In movement prediction using multiple linear regression, acquiring accurate weight matrices is important. The velocity information acquired from an accelerometer is used as reference data and is the most critical factor for accurate multiple linear regression. However, the error accumulates during the integration process because of the error of the accelerometer. Accelerometers with higher accuracy can yield better results.

The third factor is the limitation of linear regression. Obtaining a highly complicated relationship between the neural signal and hand trajectory based only on the linear model is not easy. More complex methods such as kernel regression should demonstrate a more accurate result.

### Solution

The success rate for grasping turned out to need some improvements from a practical point-of-view. Even though the success rate for grasping was not sufficiently high, the robot was able to approach the target very closely in most of the trials, and more than half of the trials resulted in touching. Even though, various methods can be used to resolve the accuracy issue, using additional sensors may be a powerful alternative considering the results of previous studies. Onose et al. [24] used a pair of cameras to find the 3D gaze point and succeeded in reaching and grasping objects with EEG. Kim et al. [39] controlled a robot arm with the assistance of external sensors and greatly improved the performance of the reaching and grasping motions. They were the first to apply continuous shared control (CSC), which was initially proposed to help humans intervene in autonomous tasks executed by a robot [40], to a BMI. Kennel et al. used a stereo camera with a robot arm controlled by MEG to grasp a target object [41]. Some studies have implemented BMI systems based on non-invasive neural signal assisted by external sensors in systems outside robot arms [42, 43]. Yeom et al. proposed using an image processing technique [44] and Kalman filtering [29] to improve the accuracy of the predicted hand trajectories. Thus, using external sensors, can greatly improve the non-invasive BMI system to realize accurate intuitive robot arm control for practical application.

### Possibility of a portable system

Although MEG has better spatial resolution than EEG, a MEG device is not portable and requires a magnetically shielded room. Therefore, EEG should be used for real-time operation. The results of this study showed that EEG provided comparable performance to MEG. Even though the trajectory predicted with EEG was statistically not as accurate as that with MEG, the practical difference was small. In particular, the TPE, which is the most crucial index for reaching and grasping, exhibited a difference of only 1.717 cm (see Fig. 7). The effect size also demonstrated that the difference between MEG and EEG is not critical (Cohen’s d < 0.50). In addition, the success rate for touching (see Table 4) supported the competitiveness of EEG. As mentioned in the Method section, EEG channels were distributed over the entire head, whereas MEG channels were distributed at the motor-related area. In addition, 64 channels were used for EEG, whereas 68 channels were used for MEG. Based on these factors, the EEG accuracy can be improved by locating more channels in the motor-related area. Therefore, an EEG-driven robot, which is a portable system, can potentially be realized.

### Future work

The proposed system is in the preliminary stage of development, and some issues need to be resolved for practical applications. First, it has not yet been applied to real patients. For online application to real patients, the training problem should be resolved. In this study, training was performed by using real hand trajectories that were derived with the accelerometer. Linear regression between the real hand trajectory and neural signal provides weight matrices that indicate the relationship between them. However, for practical application to patients who have lost their limb motor function, the training method used in this study cannot be applied because patients cannot move their upper limbs, so the data of the real hand trajectory cannot be obtained. Therefore, patients should train themselves to adapt and control the robot arm system through visual feedback. Nevertheless, the offline verification performed in this study plays an important role as a previous study on online use. Training based on visual feedback for practical applications can take a long period of time. A recent study on invasive neural signals showed that accurate control can take over 13 weeks [12]. The offline verification conducted in this study can provide reference for the application of a non-invasive BMI system to real patients. As a next step in this study, the proposed system will be applied to healthy subjects without real hand movement. Finally, it will be applied to patients with neuromuscular disorders.

The second issue is that orientation control has to be implemented. In this research, the three-DOF orientation was fixed as a constant value. In recent studies on robot arm control based on invasive neural signals [11, 12], the three-DOF translational movement, three-DOF orientation, and one-DOF grasping motion are controlled by the neural signal. However, controlling all seven DOFs in BMI systems using non-invasive neural signals is difficult because of the relatively low spatial resolution. The application of the external sensors discussed above will be helpful to resolving this issue.

## Conclusions

In this research, an IRAGS driven by 3D hand trajectories predicted from non-invasive neural signals was implemented, and reaching and grasping motions were performed for verification. The verification was conducted offline. MEG and EEG were used as the input signals. In the verification, the robot approached the target very closely in most cases, but the grasping success rate was not very high because the non-invasive neural signal is less accurate. Therefore, further improvements will be needed for the system to be suitable for practical application.

Even though MEG showed statistically better accuracy than EEG, the practical difference and effect size were not large. Furthermore, EEG used fewer channels despite them not being confined to the motor-related area. Based on these factors, the performance with EEG is comparable to that of with MEG.

In conclusion, hand arm trajectories were reconstructed with relatively high accuracy by using MEG and EEG. Additionally, the robot arm was operated by using 3D hand trajectories predicted from MEG and EEG, respectively. Even though it was not accurate enough, sufficient possibility for practical application with further improvement was shown. Furthermore, it was confirmed that performance of EEG, which enables a portable system, is comparable to that of MEG. Even though the verification in this study was performed in offline, it will contribute to practical application as a prior research.

## Abbreviations

BMI: brain–machine interface
IRAGS: integrated robot arm-gripper system
3D: three-dimensional
DOF: degrees of freedom
ECoG: electrocorticography
EEG: electroencephalography
MEG: magnetoencephalography
SSVEP: steady-state visual evoked potential
tSSS: spatiotemporal signal space separation
ERD: event-related desynchronization
IRB: Institutional Review Board
RMSE: root mean square error
TPE: terminal point error
CSC: continuous shared control
